# Reimbursement cuts and changes in urologist use of androgen deprivation therapy for prostate cancer

**DOI:** 10.1186/s12894-015-0020-y

**Published:** 2015-04-03

**Authors:** Vahakn B Shahinian, Yong-Fang Kuo

**Affiliations:** Department of Internal Medicine, University of Michigan, 1415 Washington Heights, Room 3627, SPH I, Ann Arbor, MI 48109-2029 USA; Department of Internal Medicine, University of Texas Medical Branch, Galveston, TX USA; Sealy Center on Aging, University of Texas Medical Branch, Galveston, TX USA; Department of Preventive Medicine and Community Health, University of Texas Medical Branch, Galveston, TX USA

**Keywords:** Androgen deprivation, Prostate cancer, Reimbursement

## Abstract

**Background:**

We examined the impact of urologist academic affiliation on use of androgen deprivation therapy (ADT) for prostate cancer before and after major reimbursement cuts for ADT in hopes of better understanding the influence of financial incentives on its use. In particular, we hypothesized that if financial incentive was the predominant factor driving use, we should see a narrowing in the previously documented gap of ADT use between non-academic and academic urologists following the reimbursement cuts.

**Methods:**

With the Surveillance, Epidemiology and End-Results (SEER)-Medicare linked database we examined use of ADT for potentially inappropriate indications (primary therapy of localized, lower risk tumors) among patients of 2214 urologists over the period 2000–2002 and 2004–2007, representing eras before and after reimbursement cuts. Multi-level logistic regression models were used to estimate the likelihood of ADT use adjusted for patient, tumor and urologist characteristics (academic affiliation, board certification, years in practice and patient panel size).

**Results:**

Overall, ADT use peaked in 2002 at 46.6% of patients, but dropped dramatically in 2005, with a slow continued decrease through 2007 to 31.1%. A similar pattern was evident within most strata of urologist characteristics, including academic affiliation. In the multilevel model, patients of non-academic urologists had a 30% higher odds of receiving ADT than those of academic urologists in both the eras before and after the reimbursement cuts.

**Conclusion:**

A similar proportionate drop in use of ADT among both academic and non-academic urologists following reimbursement cuts suggests that factors other than financial incentives may have played a role.

## Background

Androgen deprivation therapy (ADT), either in the form of gonadotropin-releasing hormone (GnRH) agonists or orchiectomy, has long been used for palliation of metastatic prostate cancer [1,2]. Starting in the 1990s, there was an explosive growth in use of ADT with a broadening of its application to earlier stages of disease [3]. Although this was driven in part by clinical trial evidence of benefit in some settings, such as adjuvant ADT together with radiation therapy for high-risk prostate cancer [4], there have been concerns about possible financial motivations for its use. Medicare reimbursement policies made administration of GnRH agonists very profitable (due to large discounts in the acquisition price of the drugs by physicians as compared to reimbursement) [5], and we have previously shown that patients of non-academic urologists (who are more usually paid fee-for-service) were 60% more likely to receive ADT in settings of uncertain benefit than patients of academic urologists (who are more likely salaried) [6]. More recently in 2005, Medicare reimbursement policy changes related to the Medicare Modernization Act in 2003 cut payment for GnRH agonists by over 50%, which was followed by substantial reductions in use of ADT [7]. Although these findings suggested that financial incentives played an important role, other explanations were also possible. For example, over the same period, there was growing recognition of potentially serious adverse effects related to ADT [8-10]. We therefore examined the impact of urologist characteristics on use of ADT over the period of the reimbursement cuts, with the objective of further clarifying the factors driving the reductions in its use. In particular, we hypothesized that if financial incentive was the sole, or predominant, factor, we should see a narrowing in the gap of ADT use between academic and non-academic urologists following the reimbursement cuts.

## Methods

The study protocol was approved by the local institutional review board at the University of Michigan.

### Data sources

#### Surveillance, Epidemiology, and End Results (SEER)-Medicare

The SEER program consists of a group of population-based tumor registries in selected geographic areas of the US [11]. It covered 14% of the US population until 2001, and 26% thereafter. Medicare is a federal program that covers health services for 97% of persons aged 65 years and older. The information in the two programs has been linked. The SEER-Medicare database also contains the Hospital file, which includes information on hospital characteristics such as academic affiliation and is derived from the Provider of Service survey submitted by hospitals to Medicare. The SEER-Medicare database version used for this study contains Medicare claims through 2008 and cancer cases from SEER through 2007.

### American Medical Association (AMA) Physician Masterfile

The AMA Physician Masterfile collects information on all physicians in the US, regardless of membership in the AMA [12]. The information is collected from primary sources, such as medical schools, residency training programs, state licensing agencies, and the American Board of Medical Specialties. Physicians are also surveyed every 3 years regarding their current practice.

### Study subjects

Men with incident prostate cancer from 2000 through 2002, and from 2004 through 2007, (men diagnosed in the year 2003 were excluded for reasons described below in the Study Variables section) who were at least 66 years old at diagnosis (n = 191,990) were initially selected. To ensure complete information, patients not enrolled in both Medicare Part A and Part B for 12 months before and 6 months following their cancer diagnosis (n = 19160), who died within 6 months of diagnosis (n = 3527), were members of an Health Maintenance Organization (n = 47833), or diagnosed by autopsy or on a death certificate (n = 2388) were excluded. The main interest for this study was the examination of ADT use for potentially inappropriate indications given the large changes in such use noted after the reimbursement cuts [7] and known variations in rates of such use as a function of urologist characteristics [6]. We previously defined this group as men diagnosed with clinically localized (T1 or T2), low to moderate grade (Gleason 2 to 7) tumors, who did not receive treatment with radiation or radical prostatectomy. Use of ADT in that context would be potentially inappropriate given there is no clinical trial evidence supporting its efficacy as primary therapy [13], and even under theoretical considerations, it is difficult to show survival benefit from any intervention in such patients, due to the slow natural progression and competing risk of death from causes other than prostate cancer [14]. This led to the exclusion of an additional n = 91913 patients (in whom ADT use would have been categorized as either discretionary or appropriate), leaving n = 27169 eligible patients for the main analysis.

Physicians providing care to patients within a year of diagnosis were initially identified through encrypted Unique Physician Identifier Numbers (UPINs) on Medicare physician claims, as previously published [15]. Briefly, UPINs were linked to the AMA Physician Masterfile and only physicians with urology as their primary specialty code were selected. Patients who did not see at least one urologist in the year after diagnosis on at least two different days were excluded. If a patient saw 2 or more urologists, they were assigned to the urologist with ≥75% of urologist visits in the year after diagnosis. If no single urologist accounted for ≥75% of the visits, the patient was excluded.

### Study variables

Patient demographic and tumor characteristics were derived from the SEER records in the linked database and used to categorize patients by age, ethnicity, SEER region of residence at the time of diagnosis, year of diagnosis, clinical stage (T1 through T4), and grade (low - Gleason 2–4; moderate - Gleason 5–7; poor - Gleason 8–10). Stage was assigned using the SEER Extent of Disease-Clinical Extension classification system prior to 2004 and using the Collaborative Stage Clinical Extension system from 2004 onwards [7]. Cancer grade was categorized only as a range as low (Gleason 2–4), moderate (Gleason 5–7) or high (Gleason 8–10) prior to 2003, but in 2003 only Gleason 7 was switched to the high grade category. From 2004 onwards, individual Gleason scoring has been available, allowing classification into similar groupings as for the data prior to 2003. To ensure comparability across the study period men with incident prostate cancer in 2003 were therefore excluded. The socio-economic characteristics of each patient were based on the percent of adults with less than 12 years of education and median income of the zip code of residence from the 2000 United States Census data. Comorbidity was measured using an adaptation of the Charlson Comorbidity Index for use with Medicare physician claims data, based on the period one year prior to diagnosis of prostate cancer [16,17].

Urologist board certification was available from the AMA based on information from the American Board of Medical Specialties [12]. Patient panel size was defined as the number of patients with incident prostate cancer (regardless of ADT use) assigned to each urologist over the entire study period, and categorized as <14, 14–44, 45–74, and ≥75 patients. The categorization used was similar to an approach we utilized in a previous publication, chosen to ensure a reasonable distribution for the number of patients, with cut-offs roughly corresponding to the 2nd quartile, 3rd quartile and 90th percentile [6]. Hospital academic affiliation was available from the SEER-Medicare Hospital file. Hospitals with a major academic affiliation were those that played an important role in the teaching program of a medical school. Hospitals with a minor academic affiliation had academic involvement limited to hosting of a residency program or occasional medical student rotations [6]. Urologists were categorized as having an academic affiliation if all their inpatient Medicare claims submitted were from a hospital with major or minor academic affiliation. These urologists would be more likely to be salaried, through employment with a medical school, or hospital. All other urologists were categorized as having no academic affiliation. These urologists would be more likely to be in private practice or part of single specialty groups, and therefore derive most of their income from fee-for-service activities. In initial analyses of academic affiliation using three categories (major, minor or none) patterns of ADT use were essentially identical between urologists with major and minor academic affiliation. Therefore, to optimize statistical power the main analyses were presented using a binary classification: major or minor academic affiliation vs. none.

The outcome was receipt of androgen deprivation. Androgen deprivation was defined as the receipt of at least one dose of a GnRH agonist (identified through Medicare claims codes used to designate each dose given of injectable medications [8]) or orchiectomy (defined by the presence of the Current Procedural Terminology codes or International Classification of Diseases, 9th revision [ICD-9] procedure codes in the Medicare claims) in the first six months following diagnosis of cancer.

### Statistical analyses

Differences across strata of urologist characteristics in the proportion of patients receiving ADT were tabulated. The effect of urologist characteristics on the outcome of use of androgen deprivation was evaluated using multilevel logistic regression models to account for clustering of patients within urologists. Models entering the urologist, patient and tumor characteristics listed above as independent variables were estimated. Odds ratios (OR) for the use of androgen deprivation for each urologist characteristic were calculated along with 95% confidence intervals (CI). As the main interest was to examine how the effect of urologist characteristics changed related to reimbursement cuts, the model results were stratified into two eras, 2000–2002 (prior to the reimbursement cut) and 2004–2007 (following the reimbursement cut). To specifically assess whether there were significant changes in the effect of urologist characteristics on use of ADT over the course of the two eras, tests of interaction were performed between each urologist characteristic and era, on the outcome of ADT use. In addition, the median urologist rates of androgen deprivation use as a function of academic affiliation were estimated from the models, and plotted by calendar year of diagnosis to show change in use over time. Analyses were performed with SAS version 9.3 (Cary, NC). All tests of statistical significance were two sided, with P values of less than .05 being considered significant.

## Results

A total of 2,214 urologists were identified as providing care to patients with incident prostate cancer over the study period. A majority of urologists were board certified (77.9%), had either a major or minor academic affiliation (66.2%), and were male (97.5%). Trends over time in the proportion of patients receiving ADT for inappropriate indications are presented in Table 1, stratified by urologist characteristics. In the overall cohort, such use peaked in 2002 at 46.6% of patients, but dropped dramatically in 2005, with a slow continued decrease through 2007 to 31.1%. A similar pattern was evident within most strata of urologist characteristics. Patients of older urologists, those practicing longer, and those without an academic affiliation tended to have higher rates of ADT use.

**Table 1:** **Proportion of patients receiving androgen deprivation therapy for inappropriate indications by year, stratified by urologist characteristics**

**Urologist characteristics**	**Categories**	**Urologist sample**		**ADT use for inappropriate indications by year**						
		**N**	**%**	**2000**	**2001**	**2002**	**2004**	**2005**	**2006**	**2007**
Total		2214	100	44.2	44.3	46.6	44.7	36.2	33.4	31.1
Age (years):	<45	694	31.3	40.9	45.4	48.2	40.4	33.5	30.2	29.6
	45 - 54	630	28.5	44.2	44.1	44.4	46.0	37.8	32.4	30.2
	55 - 64	672	30.4	45.2	42.4	46.1	45.7	35.4	35.0	32.5
	≥65	218	9.8	48.7	49.2	55.1	45.6	38.9	41.7	35.5
Sex:	Female	55	2.5	38.5	44.4	26.9	31.8	33.3	34.4	34.5
	Male	2159	97.5	44.3	44.3	46.7	44.8	36.2	33.4	31.1
Years in practice:	<15	864	40.1	39.7	43.2	46.1	41.7	33.5	30.8	28.4
	15 - 24	548	25.4	43.5	42.6	44.0	46.3	39.9	32.9	33.2
	25 - 34	488	22.6	47.6	44.5	47.9	44.4	33.5	35.4	31.3
	≥35	255	11.8	48.1	47.1	53.0	46.4	40.7	40.7	34.0
Board certification:	Yes	1725	77.9	44.6	44.1	46.0	44.2	36.1	33.5	30.9
	No	489	22.1	42.7	44.9	48.8	46.4	36.8	33.0	31.9
Academic affiliation:	Major or Minor	1338	66.2	41.5	43.2	44.7	41.9	33.3	31.9	28.9
	None	684	33.8	47.6	46.0	49.0	48.2	40.1	35.5	33.6
Patient panel size (no. of patients):	<14	685	30.9	37.7	44.7	42.1	46.1	34.7	35.6	29.2
	14 - 44	603	27.2	39.8	40.6	47.3	43.1	36.7	32.0	30.1
	45 - 74	452	20.4	47.2	48.2	47.2	44.9	37.8	33.4	36.0
	≥75	474	21.4	47.6	43.7	49.3	44.6	35.6	32.9	29.0

Table 2 presents multilevel models showing the odds of ADT use for inappropriate indications as a function of urologist characteristics, stratified by era of use (2000–2002 vs. 2004–2007). The only significant finding was an increase in the odds of ADT use for patients of non-academically affiliated urologists (vs. urologists with either a major or minor academic affiliation), which was similar in both eras. None of the tests for interaction between era and urologist characteristics on the outcome of ADT use were significant.

**Table 2:** **Odds of ADT use in multilevel models as a function of urologist characteristics, stratified by era**

**Urologist characteristics**	**ADT use by era**				
	^**a**^ **2000-2002**		^**a**^ **2004-2007**		
	**Odds ratio**	**95% CI**	**Odds ratio**	**95% CI**	**p-value interaction with era**
Years in practice (per 5 years)	1.01	(0.97, 1.05)	1.03	(1.00 ,1.07)	0.47
Board certification					
Yes	ref		ref		
No	1.01	(0.82, 1.24)	1.06	(0.89, 1.28)	0.94
Academic affiliation					
Major or minor	ref		ref		
None	1.32	(1.17, 1.56)	1.34	(1.15, 1.56)	0.68
Patient panel size					
<14	ref		ref		0.13
14 - 44	1.02	(0.84, 1.25)	0.93	(0.77, 1.12)	
45 - 74	1.23	(0.99, 1.54)	1.03	(0.84, 1.26)	
≥75	1.10	(0.86, 1.40)	0.89	(0.71, 1.11)	

^a^Based on multilevel model with patient age, comorbidity, ethnicity, SEER region, tumor stage, grade, year of diagnosis, census tract education, and census tract poverty entered as “level 1” variables and urologist characteristics entered as “level 2” variables. Urologist sex was not entered into the models due to low female sample size. Urologist age was not entered into the models due to collinearity with the years in practice variable.

**Figure 1:**
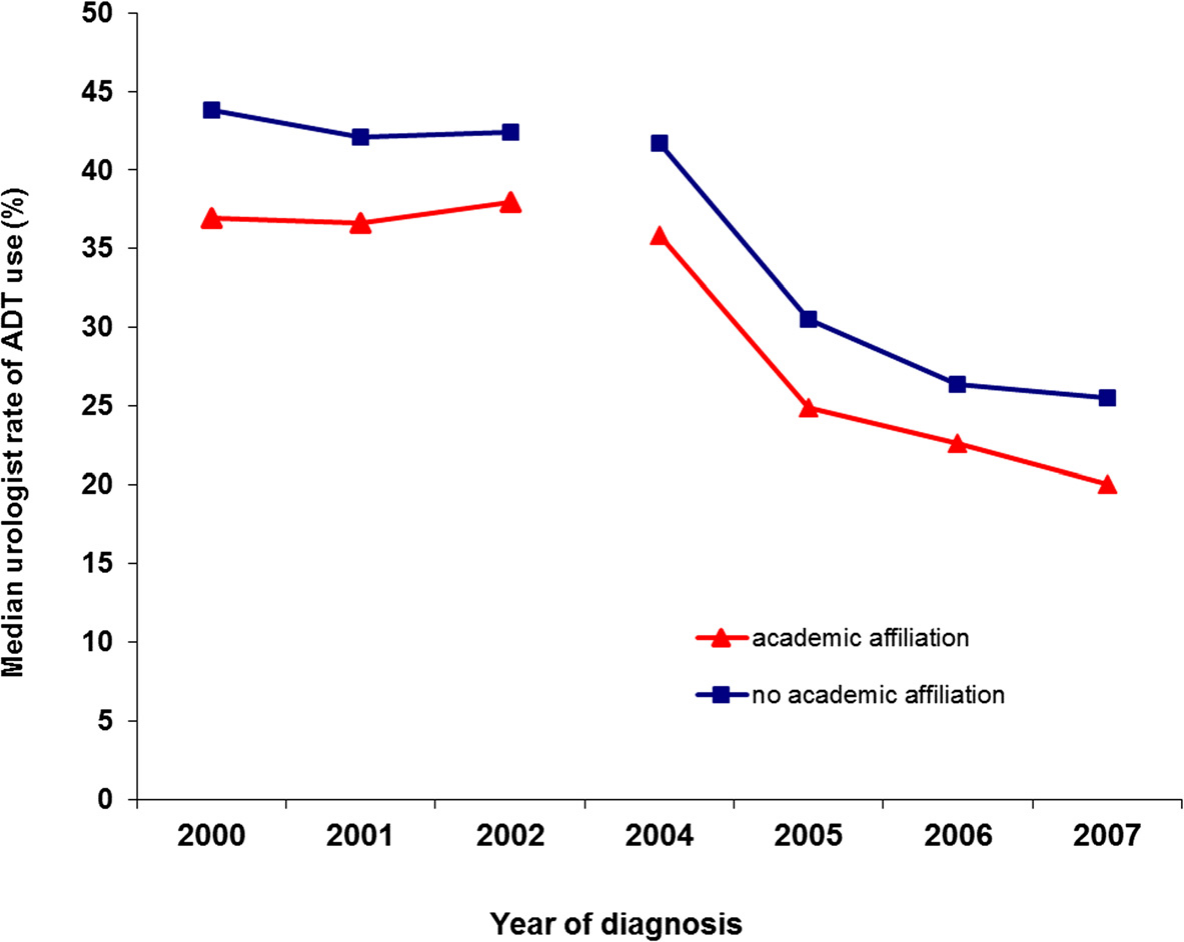
**Median urologist rate of androgen deprivation (ADT) for inappropriate indications, based on the multilevel model from Table** 2 **, by calendar year of diagnosis and stratified by urologists with academic (major or minor) versus no academic affiliation.**

The Figure 1 shows a plot of median urologist rates of ADT use for inappropriate indications over time for patients of urologists with either a major or minor academic affiliation versus urologists without an academic affiliation. Among both groups, there was a drop in use starting in 2004, but the gap between them remained relatively constant throughout the study period.

## Discussion

This study shows a drop in use of ADT for inappropriate indications following reimbursement cuts for GnRH agonists in 2004 and 2005, which occurred among patients of both academic and non-academic urologists. Notably, although overall rates of use were higher among non-academic urologists, the gap between them and academic urologists remained constant from the period before through the period after the reimbursement cuts.

We initially argued that if the reductions in use of ADT were purely financially motivated, we would expect to see a narrowing in the gap between academic and non-academic urologists as the latter would be most affected in a direct manner by cuts in reimbursement for GnRH agonists. Our findings were not consistent with such a pattern, suggesting that factors other than financial incentives may have also played a role. In support of this, previous work done in other health care contexts in which financial incentives were absent has also demonstrated a decrease in use of ADT over a similar period [18,19]. A Canadian study examining the Ontario province-wide database showed a slow but steady decline in the rate of new ADT initiation starting in the late 1990s and continuing through 2005 [19]. Most of that reduction was for use of ADT as a primary therapy. In a US study utilizing data from the Veterans Health Administration, pharmacy claims for use of GnRH agonists declined 16.8% from 2004 to 2007 [18]. However, the data presented was limited to counts, without examination of rates, or indications for use. The main competing explanation for the recent reductions in use of ADT is the concern for adverse effects. Recognition of the impact of ADT on quality of life, anemia and osteoporosis was evident as early as the 1990s [20-23]. However, it was not until 2005 and later that a series of high impact publications demonstrated potentially life threatening consequences such as fractures, cardiovascular disease and diabetes mellitus, which may have strongly discouraged use of ADT in settings without clear evidence of benefit [8-10]. Ultimately it is likely that a number of factors in combination led to the observed findings in our study, with contributions from both concerns over adverse effects as well as reduced financial incentives.

The persistent gap in use as a function of academic affiliation even after much of the financial incentive was removed may relate to differences in the interactions between urologists and patients. The pressure to provide some sort of treatment in the face of a cancer diagnosis may be a strong factor in the decision to use ADT in place of watchful waiting for patients with localized prostate cancer who are otherwise not good candidates for radiation treatment or radical prostatectomy. Physicians in academic settings may have more time for discussion of risks and benefits, leading patients to choose more conservative courses of action [24]. However, no studies have directly examined whether discussion between patients and urologists about ADT differ between academic versus non-academic urologists.

There are limitations to this study. Only men 66 years and older were included, and use of androgen deprivation in health maintenance organizations could not be examined. Assignment of urologist academic affiliation, based on Medicare claims, may have been imperfect. However, misclassification would tend to bias the results to the null, so that significant associations should still be valid. The statistical power to detect interactions between era and the effect of urologist characteristics on ADT use may have been limited. Nevertheless, inspection of the point estimates reveals no obvious differences in effect between the two eras, making it less likely that an interaction was missed due to insufficient sample size. Finally, due to limitations in the granularity of clinical detail available in a claims-based analysis, not all ADT use deemed inappropriate by our study definition is necessarily unreasonable. However, our main intention was to examine differences in ADT use between academic and non-academic urologists, which we felt (and had previously demonstrated) would be most prominent in settings where evidence of efficacy was most lacking.

## Conclusion

In conclusion, although the study findings do not rule out a role of financial incentive in the observed patterns of ADT use, they suggest that the explanation is likely to be more complex.

## Abbreviations

ADT: Androgen Deprivation Therapy
AMA: American Medical Association
GnRH: Gonadotropin-releasing hormone
SEER: Surveillance Epidemiology and End Results
UPIN: Unique Physician Identifier Number
